# Evolving Toward Subject-Specific Gait Rehabilitation Through Single-Joint Resistive Force Interventions

**DOI:** 10.3389/fnbot.2020.00015

**Published:** 2020-03-12

**Authors:** S. Srikesh Iyer, Joel V. Joseph, Vineet Vashista

**Affiliations:** Human Centered Robotics Lab, Indian Institute of Technology Gandhinagar, Gandhinagar, India

**Keywords:** gait rehabilitation, wearable robotics, cable-driven robots, subject-specific paradigm, single joint intervention

## Abstract

Walking is one of the most relevant tasks that a person performs in their daily routine. Despite its mechanical complexities, any change in the external conditions that applies some external perturbation, or in the human musculoskeletal system that limits an individual's movement, entails a motor response that can either be compensatory or adaptive in nature. Incidentally, with aging or due to the occurrence of a neuro-musculoskeletal disorder, a combination of such changes including reduced sensory perception, muscle weakness, spasticity, etc. has been reported, and this can significantly degrade the human walking performance. Various studies in gait rehabilitation literature have identified a need for the development of better rehabilitation paradigms and have implied that an efficient human robot interaction is critical. Understanding how humans respond to a particular gait alteration can be beneficial in designing an effective rehabilitation paradigm. In this context, the current work investigates human locomotor adaptation to resistive alteration to the hip and ankle strategies of walking. A cable-driven robotic system, which does not add mobility constraints, was used to implement resistive force interventions within the hip and ankle joints separately through two experiments with eight healthy adult participants in each. In both cases, the intervention was applied during the push-off phase of walking, i.e., from pre-swing to terminal swing. The results showed that subjects in both groups adopted a compensatory response to the applied intervention and demonstrated intralimb and interlimb adaptation. Overall, the participants demonstrated a deviant gait implying lower limb musculoskeletal adjustments as if to compensate for a hip or ankle abnormality.

## 1. Introduction

A major portion of an individuals daily routine involves activities involving the lower limbs, such as standing, walking, and running (Rose and Gamble, 1994; Rodgers, 1995). To perform these actions, a healthy human exhibits efficient coordination among various components of the lower limb musculoskeletal structure. Standing upright, walking, or running represent a state of fall and unbalance to which a human reacts, accounting for the anatomical joint structure and redundant muscles, to either hold the body's center of mass (COM) within an area of foot support or to advance the COM in a repetitive and controlled manner out of the area of foot support. Despite the complex mechanical structure of legs, which manifest a coupled dynamic, a healthy human adapts to accomplish these actions in the most efficient manner under varying conditions (Voloshina et al., 2013; Svoboda et al., 2016).

Incidentally, with aging or due to the occurrence of a neurological disorder, such as stroke, human performance while performing these actions degrades significantly (Ko et al., 2010; Carmo et al., 2012; Lauziere et al., 2014). Remarkably, there exists the potential to improve deviation from normal gait through proper rehabilitation methods. Conventional therapies for gait rehabilitation involve motor learning techniques by means of repetitive training (Pohl et al., 2007). Due to the laborious nature of these therapies, robotic devices have been developed in the community where the focus is to aid physical therapists in the gait training and to provide an objective measurement of gait performance. In the last decade, body-weight support systems (Duncan et al., 2011; Mao et al., 2015) and robotic leg exoskeletons (Jezernik et al., 2003; Kawamoto et al., 2003; Banala et al., 2007; Veneman et al., 2007; Goffer and Tamari, 2013; Wang et al., 2015; Bionics, 2017) have also been developed to design and implement novel external interventions to improve the rehabilitation process. The majority of these exoskeletons make use of rigid links as part of their design to support the high joint torque requirements during walking. However, the robotic systems based on rigid links have an issue of human–robot joint misalignment, are heavy, and tend to restrain the natural motion of the leg. Using such devices for rehabilitation would also entail human adaptation to undesirable mobility alterations. Notably, recent works in the community have been directed toward understanding and categorizing the human–robot joint misalignments and the compensation strategies (Näf et al., 2018). Furthermore, non-linear control methodologies are being developed and tested with lower limb exoskeletons (van der Kooij et al., 2008; Rea et al., 2014) and prostheses (Zhao et al., 2017; Khan et al., 2018) to compensate for the effect of such misalignments. Alternatively, cable-driven systems that are lightweight, flexible, and do not interfere with the natural gait have been used to develop wearable systems for gait rehabilitation. Some of these systems include C-ALEX (Hidayah et al., 2018), A-TPAD (Vashista et al., 2014), and exosuits (Zhang et al., 2017; Bae et al., 2018; Kim et al., 2018) for lower limbs.

The design goal of a robotic exoskeleton is to assist or resist the deviant human lower limb joint motion to ensure that the leg moves along the desired foot trajectory repeatedly, typically in a way to emulate a healthy gait. To achieve this, a leg exoskeleton is modeled and controlled as a multi-joint serial chain robotic system, which applies external forces on the thigh and shank segments to administer sufficient hip and knee joint torques to attain a healthy gait pattern. Studies using different leg exoskeletons have reported adaptation in the gait pattern of healthy and disabled individuals to the applied forces (Duschau-Wicke et al., 2009; Krishnan et al., 2012; Srivastava et al., 2016; Hidayah et al., 2018). These findings imply some level of adjustments by the human musculoskeletal system in response to the applied external forces on the leg. Consequently, there is a strong case for the use of robotic devices for gait training among the rehabilitation community. However, various studies have highlighted the need for further research into robotic devices, especially to the improvement of the human–robot interaction for effective gait training (Belda-Lois et al., 2011; Iosa et al., 2011; Morone et al., 2011). As external forces are applied by a robot during gait training, the understanding of human locomotor adaptation to controlled primitive external forces is particularly essential. Such modalities can be used to establish the effect of external forces in a leg exoskeleton-based gait rehabilitation paradigm to correct a deviant gait.

The major focus of any gait rehabilitation paradigm is to improve walking performance. Typically, the walking speed, gait symmetry, joint range of motion, and risk of falls are used as the walking performance measures. In particular, muscle weakness and spasticity in individuals with neurological disorders, such as stroke, result in reduced joint range of motion, reduced weight bearing on the paretic leg, and asymmetric spatiotemporal gait parameters (Carmo et al., 2012). Studies have reported that, post-stroke (Lauziere et al., 2014), there is a lack of hip flexion moment from midstance to the late stance of the paretic limb. Furthermore, due to limited paretic ankle push-off capability, post-stroke patients demonstrate compensatory kinematic strategies, such as higher hip abduction and hip circumduction along the paretic limb. In addition, with aging and muscle weakness, individuals rely upon proximal-to-distal muscle sequencing, utilizing an uneconomical hip strategy to counteract external perturbations (Mueller et al., 1994; Turns et al., 2007; Lewis and Ferris, 2008). Thus, the impaired walking performance of an individual, i.e., slow walking speed, asymmetric gait pattern, and increased risk of falls, is the collective effect of incurred alterations in the neuro-musculoskeletal system. Notably, prior understanding of lower limb musculoskeletal adjustments to gait abnormalities can be useful in adapting applied forces during a robotic gait rehabilitation paradigm.

Furthermore, the effectiveness of a robotic rehabilitation paradigm is dependent on the human response to the applied human–robot interaction, which incorporates a deviant but coupled dynamical musculoskeletal system of a human and a mechanical robotic platform. To this end, the focus of the current work is to study human locomotor adaptation resulting from external force interventions, which are applied about a single joint to resist the joint motion during walking to induce deviations in the gait pattern. A healthy individual employs a combination of ankle and hip strategies while walking. The hip strategy involves pulling the leg forward using hip flexor muscles, and the ankle strategy involves the leg being pushed forward using plantar flexor muscles (Lewis and Ferris, 2008). The ankle joint is employed to get into the swing phase, and the hip joint is involved to move the leg forward through the swing phase. People tend to use the hip strategy solely when the muscles supporting the ankle joint become weaker (Mueller et al., 1994). In stroke-survivors, the ankle plantar flexors become impaired, resulting in a reduction in the forward propulsion force during push-off (Turns et al., 2007). Considering the role of these joints during the push-off phase of walking, resistive force interventions were applied to the hip and ankle joints to resist their motion from the pre-swing to the terminal swing phase of walking.

A cable-driven Wearable Adaptive Rehabilitation Suit (WeARS), which can be adapted to apply forces at different joints and minimizes undesired mobility constraints on the wearer, was used to apply the force. Two sets of human experiments were conducted to study the locomotor adaptation, and eight healthy participants were used in each case. Experiment I involved resistive force application on the posterior thigh to resist the hip joint motion of the right leg, and Experiment II involved applying resistive force on the anterior side of the foot to resist the ankle joint motion of the right leg. It was hypothesized that the participants' response to the applied single-joint resistive force interventions would be as if to compensate abnormalities in the hip and ankle joint strategy so that the effect of the applied interventions would be reflected by a deviant gait pattern with intralimb and interlimb adjustments. The understanding drawn from such locomotor adaptation with primitive resistive force intervention would be useful in developing subject-specific gait rehabilitation paradigms.

## 2. Materials and Methods

### 2.1. Design of Wearable Adaptive Rehabilitation Suit (WeARS)

**Figure 1:**
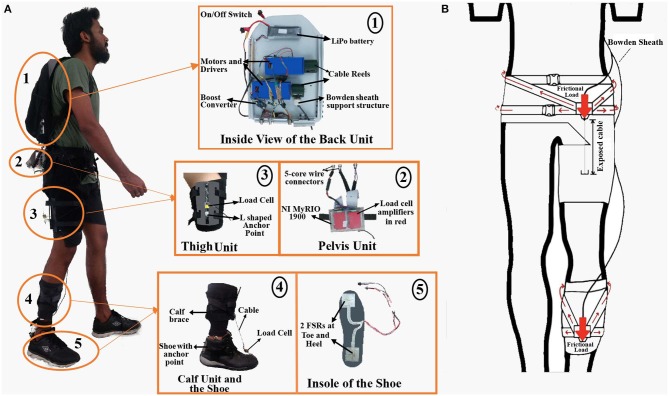
**(A)** Wearable Adaptive Rehabilitation Suit (WeARS). (1) Back unit with motors and other components mounted on the HDPE plate with aluminum frame. (2) Pelvis unit with micro-controller and amplifiers. (3) Thigh unit with anchored cables and a load cell in between to record the cable tension. (4) Calf unit with load cells and a shoe with anchor points for the cable. (5) Force Sensing Resistors (FSRs) are placed on shoe insoles to measure gait events while walking. **(B)** Frictional load distribution over the groin and calf braces is achieved using crossed nylon webbing design.

A cable-driven Wearable Adaptive Rehabilitation Suit (WeARS) was developed for implementing the force interventions as part of this work. Cables in a cable-driven system can be routed parallel to the lower limb to apply a pulling force on a segment that may either assist or resist the limb movement and can be thought of as analogous to human agonist/antagonist muscles. The schematic and component details of the setup is shown in Figure 1A. The cables make the WeARS lightweight and flexible, thus enabling unaltered leg movement; this is unlike rigid links, which can possibly induce mobility constraint. The components of WeARS are divided into the Back unit, Pelvis unit, Thigh unit, Calf unit, and Foot unit. The Back unit, the microcontroller of the Pelvis unit, and the Foot unit are the integral parts of WeARS. The Calf unit, with an attachment to a shoe, and the Thigh unit, with a groin brace, are meant be used interchangeably to apply external forces on either the ankle joint or the hip joint.

#### 2.1.1. Components

##### 2.1.1.1. Back unit

The Back unit comprises of the two geared DC motors mounted on a High Density Polyethylene (HDPE) plate housed inside a backpack. The DC motor, made by Cytron, has a torque capacity of 1.76 Nm with a rated voltage of 12V. A cable reel 4 cm in diameter was attached to the shaft of the motor for winding the cable. As the motor rotates, the cable wraps or unwraps from the cable reel to allow variations in the cable tension values. Furthermore, a battery, boost converter (XL6009), and Bowden sheath support structures made of Delrin (polyoxymethylene) are also part of the Back unit. A steel cable of 0.8 mm diameter that goes through the Bowden sheath is used to apply the force. The weight of the Back unit, 2.5 kg, is distributed through the backpack over the participant's back. The motors mounted inside the backpack generate the desired tension in the cables. These cables are routed along the leg through Bowden sheaths, which are fixed at the ends to minimize the friction in a manner similar to the braking system used in bicycles. One end of the Bowden sheath is fastened on the supporting brace (Figure 1B), and the other end is attached to the backpack plate, which helps to distribute the frictional load on the back. The Boost converter helps to step up the voltage to operate the amplifiers on the pelvis unit. Cable and wire connectors are used to connect the backpack unit with the pelvis unit.

##### 2.1.1.2. Pelvis unit

The Pelvis unit comprises a microcontroller (National Instruments myRIO 1900), amplifiers (Futek IAA100), nylon webbing straps, and Bowden sheath support structures over a flexible groin brace made of breathable Neoprene. The broad nylon webbing straps provide more surface area for load distribution while the breathable Neoprene prevents sweltering, adding to the user's convenience. The microcontroller has a built-in accelerometer placed over the pelvis, and this is used to fetch the pelvic acceleration. The Bowden sheath from the back unit is fastened on the Bowden sheath support structure on the groin brace. Thus, the frictional load between the cable and the Bowden sheath is concentrated on these support structures on the groin brace and the back unit. The frictional load is distributed over the waist region through the nylon webbing straps with tightening buckles sewn over the brace. The large arrows in Figure 1B show concentrated frictional load on the Bowden sheath support structures, and the small arrows show the distribution of frictional load over the suit. The cable from the backpack passes through the fixed Bowden sheath to be anchored on the thigh or calf unit. The groin brace allows the pelvis unit to be worn by people of distinct pelvic and thigh widths.

##### 2.1.1.3. Thigh unit

The thigh unit has an aluminum sheet with flexible Velcro straps and anchor points for the cable coming from the back unit to be attached. The anchor points are made of Delrin in an L shape, the cable is mounted on the short arm of L, and the longer arm is attached to the aluminum sheet so that the force is distributed over the thigh unit. This unit is used for hip actuation. The load cell (Futek LSB200) is placed between the anchor point and the cable coming from the back unit to record the tension in the cable. The load cell signal is sent to the microcontroller through the amplifiers, which are located in the pelvis unit.

##### 2.1.1.4. Calf unit

The calf unit has a calf brace made of breathable Neoprene with Bowden sheath support structures, and nylon webbing straps are sewn onto it. The calf unit utilizes a similar design as the pelvis unit for the distribution of friction load. The cable passes through the fixed Bowden sheath to be anchored on to the foot unit. The calf unit is used to hold the Bowden sheath in place so that the shoe with the anchor point could be used for ankle actuation.

##### 2.1.1.5. Foot unit

The foot unit consists of force sensing resistors (FSRs) mounted on a shoe insole to record the heel and toe pressure. The FSR signals are processed in real time at the microcontroller in the pelvis unit to detect heel-strike and toe-off events. Multiple such foot units of varying sizes were prepared so that they may fit the participants according to their shoe size. Special shoes of different sizes were prepared to be used along with the calf unit. These shoes have anchor points on the front and back ends to attach from the calf unit. A load cell (Futek LSB200) is located between the anchor point and the cable coming from the back unit to record the tension in the cable.

### 2.2. Control Methodology

The cables in WeARS can only apply pulling forces on the lower limb. Based on when the forces are applied during a gait cycle, they can either be assistive or resistive to the limb movement. Furthermore, there is inherent variability in the human gait, and the gait cycle duration therefore varies from one cycle to another. A human continuously adjusts the lower limb movements to accommodate these changes. Thus, to apply the desired force intervention, WeARS needs to adapt to the changes in walking frequency. The control methodology of WeARS is divided into a high-level controller and a low-level controller to allow for the subject-specific gait adaptation.

#### 2.2.1. High Level Control

**Figure 2:**
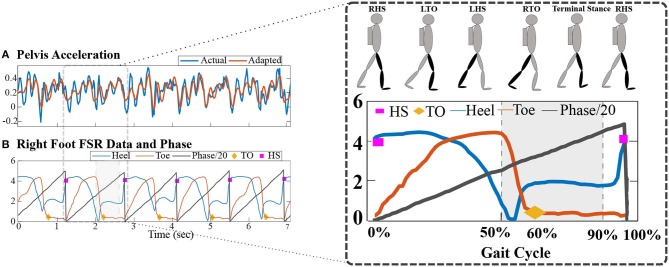
**(A)** Desired vertical pelvic acceleration and adapted pelvic acceleration profiles during a walking trial. **(B)** FSRs data from right foot with Heel-Strike (HS) and Toe-Off (TO) events. Gait phase values in percentages are calculated using Algorithm 1 and gait events. The shaded gray region shows the 50–90% of a gait cycle during which WeARS applied the force intervention.

The main goal of the high-level controller is to detect the gait phase of walking during the experiment to determine the applied force duration and profile. In this work, the synchronization properties of Adaptive Frequency Oscillators (AFOs) when coupled to a dynamical system were used (Righetti et al., 2006). As per Algorithm 1, these oscillators dynamically adapt the parameters to learn the frequency of human walking, a periodic system, in real time without any signal-processing techniques (Vashista et al., 2016). The periodic pelvic acceleration signal, *a*_*y*_, recorded using an accelerometer mounted on the pelvic microcontroller unit, was used to compute the desired acceleration value, θ_*d*_. A set of three oscillators, M, were used to calculate the adapted acceleration value, θ_*t*_, using learning factor, ν, coupling strength, ϵ, and minimum frequency limit, *f*_*min*_, set as 2, 10, and 1.3 Hz, respectively. The oscillator states, Y, consisting of phase, ϕ, frequency, ω, amplitude, α, and offset, β, were solved as a function of teaching signal, *F*_*t*_. The adapted acceleration and desired acceleration values during a walking trail are shown in Figure 2A.

**Algorithm 1:**
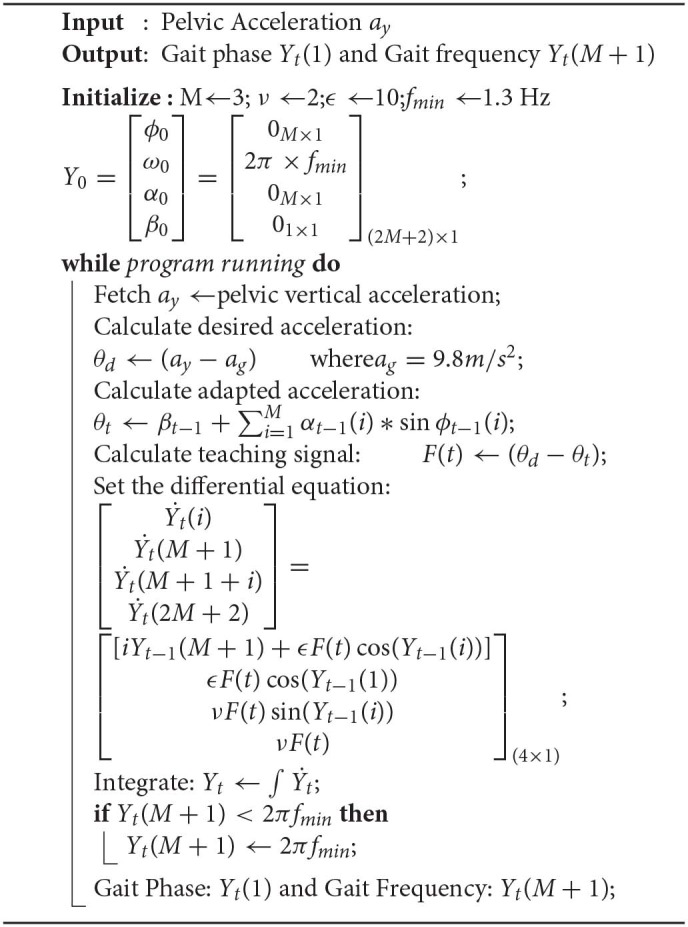
Gait Phase Adaptation

The data of force-sensitive resistors (FSRs) mounted on the foot unit insole were used to detect the gait events, Heel-strike (HS), and Toe-off (TO) (Mazumder and Vashista, 2017). To account for the variations in the walking style of different individuals, a calibration algorithm was implemented before detecting gait events. During the calibration period, five instants of maximum and minimum FSRs pressure values were averaged to determine the pressure range for the heel and toe sensors. These range values were then used to normalize the incoming signals from FSRs. As shown in Figure 2B, an HS event is detected when the normalized heel pressure value is ≥80% of the heel pressure range value, and a TO event is detected when the normalized toe pressure value is ≤5% of the toe pressure range value. The phase of the first oscillator, ϕ_1_ = *Y*_*t*_(1), from Algorithm 1 along with the knowledge of HS events of a leg gives the gait phase value in a percentage. As demonstrated in Figure 2B, the gait phase resets to 0% at each HS event, thus enabling the subject-specific gait adaptation.

**Figure 3:**
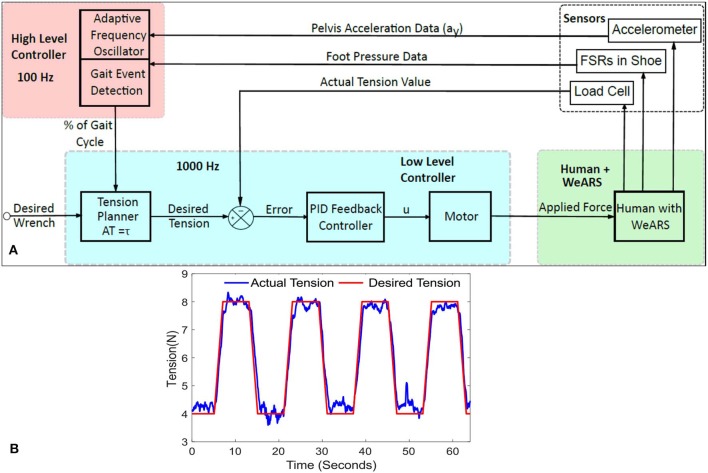
**(A)** Control architecture: the high-level controller, at 100 Hz, provides the gait phase values in %age of the gait cycle and adapts human walking using adaptive frequency oscillators and gait events. A low-level controller, at 1,000 Hz, plans the cable tension values based on desired force intervention, wrench, and uses a PID feedback control to implement the cable tension values. **(B)** PID performance response while controlling a desired cable tension value.

With the knowledge of the gait phase during walking, WeARS can apply the desired force intervention at a desired point in a gait cycle or during a desired region of a gait cycle. In addition, the controller adapts to the inherent small variations of human gait frequency. For example, the highlighted region in Figure 2B reflects 50–90% of a gait cycle, which remains invariant of changes in gait cycle time. The overall control architecture is shown in Figure 3, where the high-level controller was executed at 100 Hz.

#### 2.2.2. Low Level Control

The main goal of the low-level controller is to implement a force control to apply the desired force values. As shown in Figure 3, the low-level controller was operated at 1,000 Hz and utilized a PID-based feedback control to control the cable tension values. A cable is a unidirectional force application element, i.e., it applies only a pulling force on a body. Thus, a cable-driven system requires redundant actuation, i.e., at least *n* + 1 cables are required to control a *n* degrees of freedom (DOFs) system (Ming and Higuchi, 1994). Typically, cables in a cable-driven system are modeled as a pure force at the attachment point to evaluate the applied wrench, τ, at the end-effector using Lagrange's method (Rezazadeh and Behzadipour, 2011),

(1) $$\begin{array}{l} \text{A}T=\tau \end{array}$$

Here, **A**_(*n*×*m*)_, referred to as the structure matrix, represents the linear mapping between the cable tension values, *T*_(*m*×1)_, and applied wrench, τ_(*n*×1)_. Due to the actuation redundancy, Equation (1) comes out to be underdetermined. Several analytic and numerical optimization methods have been proposed in the literature, and a quadratic programming problem can be utilized to solve for the cable tension values (Oh and Agrawal, 2005; Sanjeevi and Vashista, 2017). As shown in Figure 3, the tension planner provides the desired cable tension values based on the applied force intervention, i.e., desired wrench.

Force control is used to drive the motors, as shown in Figure 3A. The error between the actual tension, available from the load cell, and the desired tension, calculated using the tension planner, serves as input to a PID feedback controller. The PID output, u, defines the input signal (duty cycle of PWM and direction of rotation) of motors to actuate the cables in WeARS to apply external forces on the leg segment. For the human locomotor experiments, WeARS applies a desired resistive force at the lower limb. Notably, to tune the PID controller, calibration experiments were conducted. A performance plot during such testing, where the controller follows a trapezoidal shape desired cable tension profile, is shown in Figure 3B. The PID gains values, Kp = 4.5, Ki = 1125, and Kd = 0.0009, resulted in a root mean square error (RMSE) equal to 0.362 N.

### 2.3. Experiments

Human walking is a mechanically complex phenomenon that involves coupled motion of the lower limb joints and requires cyclic activation of leg muscles. Any deviation in the gait pattern can be reflected through changes in the joint trajectories and muscles actuation patterns. In this work, we studied human locomotor adaptation due to external force interventions about a single leg joint that resists the joint motion to induce deviations in the gait pattern.

#### 2.3.1. Resistive Force Intervention

**Figure 4:**
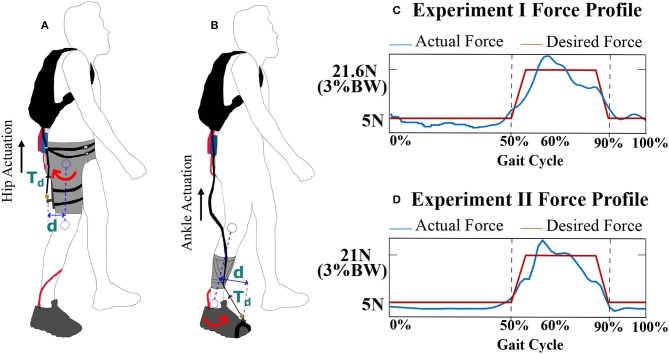
**(A)** WeARS setup for the hip actuation experiment, Experiment I, with a thigh unit. **(B)** WeARS setup for the ankle actuation experiment, Experiment II, with a calf unit and shoe with an anchor point to attach the cable. *T*_*d*_ = *Tension*, *Wrench* = *T*_*d*_ × *d*. **(C)** The desired and actual force variation during a walking trial of Experiment I for a participant with a weight of 72 kg, 3%BW equals 21.6N. **(D)** The desired and actual force variation during a walking trial of Experiment II for a participant with a weight of 70 kg, 3%BW equals 21 N. BW is body weight.

A healthy individual employs a combination of ankle and hip strategies while walking. During normal walking conditions (Rose and Gamble, 1994; Rodgers, 1995; Winter, 2009), the leg is typically in pre-swing at 50% of the gait cycle and is getting ready for toe-off. During this time, the quadriceps, the muscle group at the anterior thigh, assist in initiating the swinging of the leg through hip flexion. During the pre-swing phase, the knee joint remains extended. Around the same time, the plantar flexors, mainly the muscles in the calf, like gastrocnemius and soleus, actuate to produce propulsive force for ankle push-off. The muscle group at the posterior thigh, the hamstrings, are mainly employed to decelerate the leg during terminal swing, peaking at 95% of the gait cycle. Notably, the knee joint flexes during the swing phase, starting around 60%. Furthermore, dorsiflexors, like Tibialis Anterior, act for forefoot ground clearance during the initial swing and hold the ankle in position for the initial contact. Consequently, any external force applied to the posterior of the thigh and the anterior of the foot together or separately during the push-off phase will alter the normal muscle activity necessary for forward foot propulsion. In this work, a resistive force proportional to 3% of a participant's body weight (BW) was applied to the posterior part of the thigh of the right leg and to the anterior part of the foot of the right leg in two different experiments during 50–90% of the gait cycle, i.e., the pre-swing to terminal swing phase. The WeARS was used to implement the resistive force intervention during the desired phase of walking. Figures 4A,B show the setup that the participants wore for the hip experiment and for the ankle experiment, respectively. To apply the 3% BW force at a joint, a single cable with desired cable tension value, *T*_*d*_, was connected at the anchor point on the brace that provided an effective moment arm, *d*, to apply a resisting moment of *d* × *T*_*d*_ about the hip or ankle joint separately in the two experiments. The desired resistive force profile was such that the applied force increased from 5 N up to 3% BW till 55% of the gait cycle; here, it remained constant till 85% of the gait cycle before finally decreasing back to 5 N from 85 to 90%. A constant force of 5 N was maintained, so as to avoid cable slacking, other than at 50–90% of the gait cycle. Figures 4C,D illustrate the desired and actual force values during a walking trial of Experiment I for a participant with a weight of 72 kg and of Experiment II for a participant with a weight of 70 kg. The gray region in Figure 2B illustrates the force application phase from 50 to 90% of walking.

#### 2.3.2. Experimental Protocol

Eight healthy males in the age range 20–25 years (mean age: 22 years) and mean weight 75.25 kg (SD: 13.81 kg) participated in Experiment I. In addition, eight healthy males in the age range 20–26 years (mean age: 24 years) and mean weight 70.33 kg (SD: 14.09 kg) participated in Experiment II. Both experiments involved three sessions in the order of Baseline (BL), Training (T), and Post-Training (PT). During each session, each participant walked for 5 min at a constant speed of 3 kmph. A break of 2 min was given before T session to engage the robot and after T to disengage the robot. Data were recorded at the first, third, and fifth minute for 1 min in each session, and corresponding datasets are referred to as BL1, BL2, and BL3 during the baseline session; T1, T2, and T3 during the training session; and PT1, PT2, and PT3 during the post-training session. Each participant was suited up with reflective markers to record the human motion data via Vicon Motion Capture. For Experiment I, the participants wore the setup, as depicted in Figure 4A, consisting of thigh unit that distributes the applied force posterior to thigh of the right leg during T session. For Experiment II, the participants wore the setup, as depicted in Figure 4B, consisting of calf unit that distributes the applied force anterior to right leg's foot during T session. The term “Perturbed Leg” is used in this paper to represent the right leg on which external force was applied.

**Figure 5:**
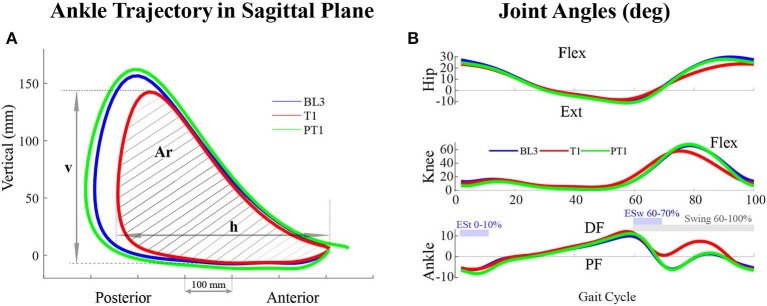
**(A)** Ankle trajectory of the perturbed leg (right leg) in the sagittal plane of walking of a participant across sessions in Experiment I. The area enclosed within the trajectory (Ar) as well as the and horizontal (h) and vertical (v) spans decreased during the training (T1) session. **(B)** Joint angles of the perturbed leg of a participant across sessions in Experiment I. During the training (T1) session, the joint angles values vary with respect to baseline (BL3) and post-training (PT1) sessions. For the hip and knee joints, Flexion, Flex, is positive and Extension, Ext, is negative. For the ankle joint, Dorsiflexion, DF, is positive and Plantarflexion, PF, is negative.

The walking kinematics was recorded using the plugin gait model in Nexus Software (Vicon Motion Systems Limited, Oxford, UK). The data were divided into gait cycles, which were defined from one heel-strike to a consecutive heel-strike. The gait events, heel-strike and toe-off, were used to compute the spatiotemporal parameters, such as stride length, stride time, step length, swing time, stance time, and single support and double support time. Stride length was defined as the distance between two successive contacts of the same foot, and ground and step length were defined as the distance between the heel of the concerned foot and the other foot at the heel-strike of the concerned foot. To avoid inter-individual variability, the spatial parameters were normalized using each subject's leg length, and the temporal parameters were normalized using $\sqrt{\frac{leg\text{\_}length}{g}}$, where *g* = 9.81 *m*/*s*^2^, following the conventions mentioned in Hof (1996). Furthermore, the joint angle trajectories were computed during all sessions, and joint parameters, such as range of motion, peak extension, and peak flexion for hip and knee joints, were computed. Peak ankle plantarflexion during early stance (ESt, defined as 0–10% of the gait cycle) and during early swing (ESw, defined as 60–70% of the gait cycle) were computed. Furthermore, peak ankle dorsiflexion values during swing (defined as 60–100% of the gait cycle) were also evaluated. To represent the motion of the leg in the sagittal plane of walking, the ankle trajectory was extracted from the markers data. Furthermore, ankle trajectory parameters, such as the horizontal span (h), vertical span (v), and the area enclosed within the ankle trajectory (Ar), were evaluated during the experiments, as shown in Figure 5A.

Statistical analysis was performed using the gait parameters data, averaged over sessions BL3, T1, T3, PT1, and PT3, to check for the significant changes between sessions within Experiments I and II. Mauchly's test of sphericity was used to test the normality condition. A one-way analysis of variance with repeated measures was carried out on these parameters, and a pairwise comparison between recordings of the sessions using the Bonferroni test was conducted for *post-hoc* analysis. All statistical analyses were performed using OriginPro software (OriginLab Corporation, Northampton, MA 01060, USA), and *p*-values of < 0.05 were considered statistically significant.

## 3. Results

### 3.1. Experiment I

With the application of a resistive force on the posterior thigh during the training session, the subjects attained a reduced ankle trajectory in the sagittal plane of walking for the perturbed leg (right leg). Plots of a representative participant in Figure 5A show a reduced area with shorter horizontal and vertical spans. Noticeable reductions were also observed in the perturbed leg's joint angle values. Figure 5B shows the hip, knee, and ankle angles of a representative participant.

The repeated measures ANOVA reported a significant difference in the values of the vertical span, v (*p* < 1*E* − 3), horizontal span, h (*p* < 1*E* − 3), and the area enclosed within the ankle trajectory, Ar (*p* < 1*E* − 3), of the perturbed leg between sessions. The Bonferroni *post-hoc* pairwise analysis reported that these three parameters, v, h, and Ar, decreased significantly during the training session compared to the baseline, BL3-T1, and retained their values during training (T1–T3 not significant) for the perturbed leg (Table 1). Furthermore, the ankle trajectory parameters were not significantly different between the baseline and post-training sessions, which implies no after-effects in these parameters. For the unperturbed leg, significant differences were observed in the values of the horizontal span, h (*p* = 0.017), and the area enclosed within the ankle trajectory, Ar (*p* = 0.02), but not in the values of the vertical span, v (*p* = 0.054). However, the *post-hoc* analysis did not report significant differences in the ankle trajectory parameters' values of the unperturbed leg between sessions.

**Table 1:** Ankle trajectory parameters for both legs, Perturbed Leg (PL) and Unperturbed Leg (UL), in Experiment I.

**Concerned leg**	**Parameters**	**T1-BL3**	**T3-BL3**	**T3-T1**	**PT1-T3**	**PT1-BL3**	**PT3-BL3**
Perturbed Leg (PL)	Ar (mm^2^)	−7 896.15^*^	−6106.33	1789.82	8 718.11^*^	2611.78	1036.77
	v (mm)	−13.95^*^	−8.9	5.05	8.71	−0.19	1.81
	h (mm)	−31.4^*^	−16.03	15.37	33.90^*^	17.87	1.74
Unperturbed Leg (UL)	Ar (mm^2^)	−1 878.46	42.33	1920.79	2567.13	2609.46	906.88
	v (mm)	−3.51	1.25	4.77	2.94	4.2	2.48
	h (mm)	−27.62	−1.64	25.98	10.76	9.12	1.11

Each column indicates the difference in means between two sessions and

* * indicates the pairwise significant difference*.

**Figure 6:**
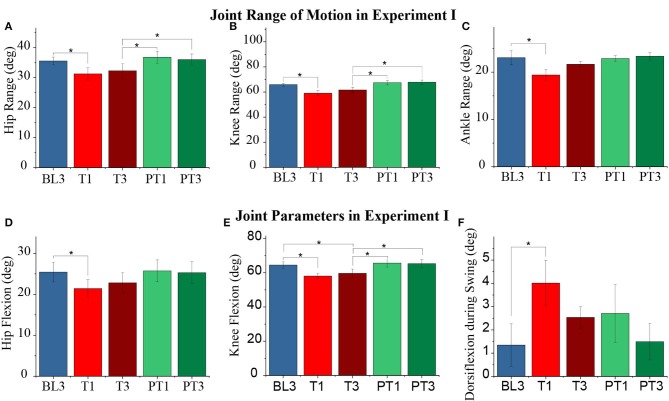
**(A–C)** Range of motion of the perturbed leg's hip, knee, and ankle joints, respectively, for different sessions. **(D–F)** Perturbed leg's peak hip flexion, knee flexion, and ankle dorsiflexion during the swing over sessions, respectively. The data presented here is averaged across all participants of Experiment I with standard error (*p ≤ 0.05).

The perturbed leg's joint range of motion changed significantly during the experiment: hip (*p* < 1*E* − 3), knee (*p* < 1*E* − 3), and ankle (*p* = 0.006), as shown in Figures 6A–C. The pairwise comparison reported a significant reduction in the hip, knee, and ankle range of motion values from baseline to training session (BL3-T1). Furthermore, these values were retained during the training session (T1–T3 not significant). The changes in the three joints' range of motion were not significantly different between the baseline and post-training sessions. Significant changes were also reported in the peak hip flexion (*p* = 0.002), peak knee flexion (*p* < 1*E* − 3), peak ankle dorsiflexion during swing (*p* = 0.03), peak ankle plantarflexion during early swing, ESw (p = 0.014), and peak ankle plantarflexion during early stance, ESt (p = 0.017) values of the perturbed leg. The pairwise comparison reported a significant reduction in the peak hip flexion and peak knee flexion values during training (BL3-T1), which was retained during the training session (T1–T3 not significant), as shown in Figures 6D,E. Notably, the changes in the ankle plantarflexion values did not report any significant pairwise comparison. Further, a significant increase was reported in the peak ankle dorsiflexion values during training (BL3-T1), as shown in Figure 6F. For the unperturbed leg, the statistics did not report any significant changes in the joint range of motion and joint parameters values.

**Figure 7:**
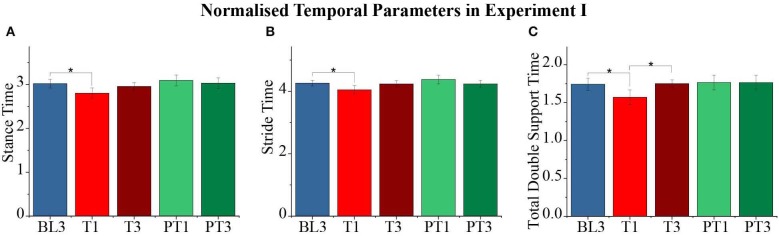
**(A–C)** Normalized stance time, stride time, and total double support time of the perturbed leg for different sessions. The data presented here are averaged across all participants of Experiment I with standard error (*p ≤ 0.05).

The effect of the resistive force was also observed in the temporal parameters of a gait cycle. The normalized values are shown in Figures 7A–C. Significant changes were reported in the stance time (*p* = 0.001) and stride time (*p* = 0.001) values of the perturbed leg and the total double support time (*p* = 0.004) during the experiment. The pairwise analysis reported a significant reduction in the stance time and stride time values of the perturbed leg and the total double support time from baseline to training (BL3-T1). During the training session, the values of the stance and stride time were retained (T1–T3 not significant) while the total double support time returned to the baseline values (T1–T3, *p* ≤ 0.05). Furthermore, the changes between the baseline and post-training sessions were not significant for the temporal measures. The double support time when the perturbed leg was leading and when the unperturbed leg was leading changed significantly (*p* = 0.04 and *p* = 0.014, respectively), but the pairwise analysis did not report statistical significance. Moreover, no significant changes were reported in the spatial parameters of both perturbed and unperturbed legs.

### 3.2. Experiment II

**Figure 8:**
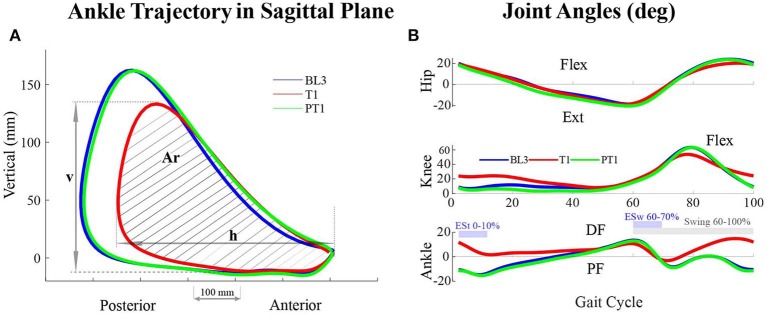
**(A)** Ankle trajectory of the perturbed leg (right leg) in the sagittal plane of walking of a participant across sessions in Experiment II. The area enclosed within the trajectory (Ar) as well as the horizontal (h) and vertical (v) span decreased during the training (T1) session. **(B)** Joint angles of the perturbed leg of a participant across sessions in Experiment II. During the training (T1) session, the joint angles values vary with respect to baseline (BL3) and post-training (PT1) sessions. For the hip and knee joints, Flexion, Flex, is positive, and Extension, Ext, is negative. For the ankle joint, Dorsiflexion, DF, is positive, and Plantarflexion, PF, is negative.

The subjects in Experiment II attained a reduced ankle trajectory in the sagittal plane of walking for the perturbed leg (right leg) when external resistive force was applied anterior to foot. A representative participant's ankle trajectory is shown in Figure 8A, which shows a reduced area with shorter horizontal and vertical spans. The hip, knee, and ankle joint trajectories of the perturbed leg also showed significant changes during the training session, as shown in Figure 8B for a representative participant. Noticeable reductions were observed in the joint angle values during the training session.

The repeated measures ANOVA reported a significant difference in the vertical span, v (*p* < 1*E* − 3), horizontal span, h (*p* = 0.017), and the area enclosed within the ankle trajectory, Ar (*p* < 1*E* − 3) for the perturbed leg. The pairwise analysis reported that the values of v and Ar decreased significantly during training compared to baseline (BL3-T1) with retention during training (T1–T3 not significant), as shown in Table 2. Notably, the reductions in the h values were not reported significant. Also, there were no significant changes between baseline and post-training sessions for the perturbed leg, which implies that there were no aftereffects in these ankle trajectory parameters. For the unperturbed leg, significant differences were observed in the h values (*p* = 0.03) but not in the v (*p* = 0.5) and Ar values (*p* = 0.2). However, the *post-hoc* analysis did not report significant differences in the ankle trajectory parameters' values of the unperturbed leg between sessions.

**Table 2:** Ankle trajectory parameters for both legs, Perturbed Leg (PL) and Unperturbed Leg (UL), in Experiment II.

**Concerned leg**	**Parameters**	**T1-BL3**	**T3-BL3**	**T3-T1**	**PT1-T3**	**PT1-BL3**	**PT3-BL3**
Perrturbed Leg (PL)	Ar (mm^2^)	−6,921.83^*^	−5,788.32	1,133.51	6,962.92^*^	1,174.6	516.3
	v (mm)	−20.16^*^	−18.45^*^	1.71	19.44^*^	0.99	0.74
	h (mm)	−25.47	−23.13	2.34	14.27	−8.86	6.23
Unperturbed Leg (UL)	Ar (mm^2^)	−975.37	−2,017.25	−1,041.88	2,016.67	−0.58	534.71
	v (mm)	0.5	−2.38	−2.93	2.34	−0.04	0.9
	h (mm)	−21.02	−18.92	2.1	11.78	−7.14	6.45

Each column indicates the difference in means between two sessions and

* *indicates the pairwise significant difference*.

**Figure 9:**
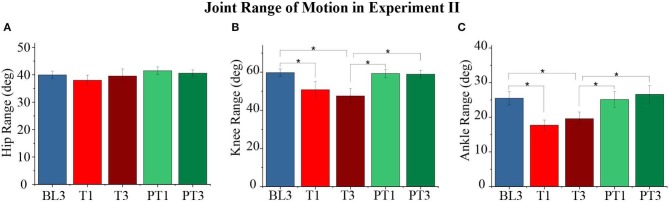
**(A–C)** Range of motion of the perturbed leg's hip, knee, and ankle joints for different sessions. The data presented here are averaged across all participants of Experiment II with standard error (*p < 0.05).

**Figure 10:**
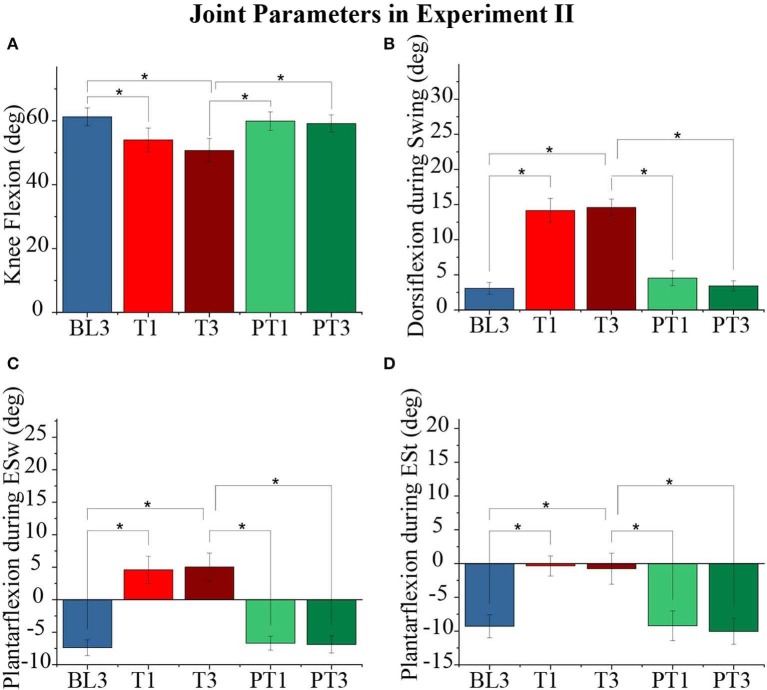
**(A)** Knee Flexion values of the perturbed leg for different sessions. **(B)** Ankle dorsiflexion values of the perturbed leg during the swing for different sessions. **(C)** Ankle plantarflexion values of the perturbed leg during early swing (ESw) for different sessions. **(D)** Ankle plantarflexion values of the perturbed leg during early stance (ESt) for different sessions. The data presented here are averaged across all participants of the Experiment II with standard error (*p < 0.05).

The effect of force training showed significant changes in the knee (*p* < 1*E* − 3) and ankle (*p* < 1*E* − 3) joint range of motion values for the perturbed leg, as shown in Figures 9A–C. The hip joint range of motion values (*p* = 0.1) did not show a significant difference. The pairwise analysis reported a significant decrease in the range of motion values for ankle and knee joints from baseline to training (BL3-T1 and BL3-T3) with retention in training (T1–T3 not significant). The reductions in the joint range of motion values during training were observed to be due to significant changes in the peak knee flexion (*p* < 1*E* − 3), peak ankle dorsiflexion during swing (*p* < 1*E* − 3), peak ankle plantarflexion during early swing, ESw (*p* < 1*E* − 3), and peak ankle plantarflexion during early stance, ESt (*p* < 1*E* − 3). These changes are plotted in Figures 10A–D. The pairwise analysis reported that the peak knee flexion, peak ankle plantarflexion during ESw, and peak ankle plantarflexion during ESt decreased significantly from baseline to training (BL3-T1 and BL3-T3). Furthermore, the peak ankle dorsiflexion during swing increased significantly from baseline to training (BL3-T1 and BL3-T3). These parameters were retained during the training session (T1–T3 not significant). The changes in the joint parameters of the perturbed leg did not show aftereffects during the post-training. Moreover, there were no significant changes in the joint range of motions and joint parameters values of the unperturbed leg.

**Figure 11:**
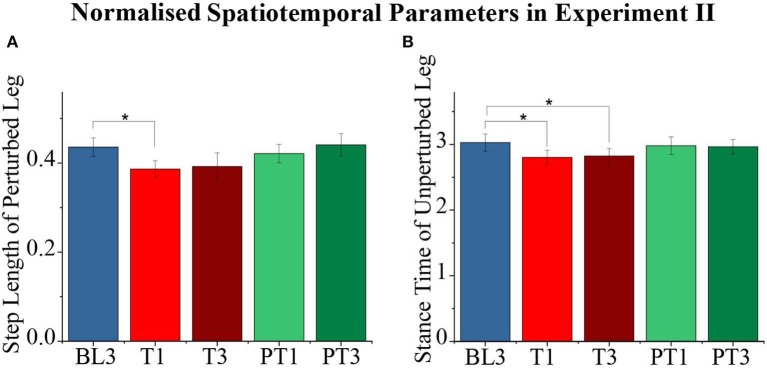
**(A,B)** Normalized step length, stance time of the perturbed leg for different sessions. The data presented here are averaged across all participants of Experiment II with standard error (*p < 0.05).

The spatiotemporal parameters during a gait cycle also reported changes due to the force training as shown in Figures 11A,B. The perturbed leg's step length (*p* = 0.001) and unperturbed leg's stance time (*p* = 0.002) showed significant difference during the experiment. The pairwise analysis reported that these values decreased significantly from baseline to training–BL3-T1 for perturbed leg's step length and BL3-T1 and BL3-T3 for unperturbed leg's stance time with retention during training (T1–T3 not significant). Furthermore, there were no significant changes between the baseline and post-training sessions. Moreover, other spatiotemporal parameters did not report significant changes during the experiment.

## 4. Discussion

Considering the significant impact gait disabilities have on an individual's independence and on society as a whole, there is an urgent need to focus on developing rehabilitation methodologies with a long-term impact. Notably, the current understanding in the field favors the use of robotic devices but identifies the need for designing a suitable human–robot interaction paradigm for effective rehabilitation (Belda-Lois et al., 2011; Iosa et al., 2011; Morone et al., 2011). Since elderly persons and people with neurological disabilities demonstrate multiple gait alterations that affect their walking performance, the focus of the current work has been to study human locomotor adaptation to controlled primitive external resistive forces. In particular, the work sought to understand how a healthy human adapts the walking performance in response to an externally applied single-joint gait alteration. A healthy individual, unlike an elderly person or an individual with lower limb disability, efficiently employs a combination of ankle and hip strategies while walking. Considering the critical role of these strategies during walking, resistive force interventions were applied either to the hip joint or ankle joint from the pre-swing to the terminal swing phase of walking.

In the current experiments, external resistive forces were applied during 50–90% of a gait cycle on the posterior side of the right thigh in Experiment I and on the anterior side of the right foot in Experiment II. The applied forces reduced hip joint flexion during the swing phase of the perturbed leg in Experiment I and reduced ankle joint plantarflexion during the early swing phase of the perturbed leg in Experiment II. In both experiments, the knee joint flexion also reduced during the swing phase of walking. During normal walking, a healthy individual utilizes the period from pre-swing to terminal swing phase of walking to generate the required propulsive force to move the leg forward with adequate foot clearance through a combination of ankle joint plantarflexion, and hip, and knee joints flexion (Rose and Gamble, 1994; Rodgers, 1995; Winter, 2009). Notably, the observed reduction in the values of knee flexion and ankle trajectory span are proxies for the reduced propulsive force generation and forward motion of the perturbed leg. Thus, any alteration to the hip and ankle joint strategy due to an external constraint can result in impaired swing initiation; this may lead to inadequate propulsion of the leg, which is the case observed in hemiparetic gait due to similar musculature abnormalities (Chen et al., 2005).

In the current experiments, the lack of significant changes in the unperturbed leg's parameters also implied an asymmetric gait adoption. Overall, the resistive nature and the duration of the applied forces on the ankle or hip joint separately induced a deviant gait in healthy participants. In contrast, the application of assistive forces at the ankle or hip joint during walking have been reported to improve the walking performance of individuals with a disability (Lewis and Ferris, 2008; Zhang et al., 2017; Bae et al., 2018; Ding et al., 2018). Importantly, the nature and duration of external forces applied to these joints during a robotic rehabilitation setting are crucial for effective gait training.

Locomotor adaptation is the process of adapting one's motor response with practice due to an induced error. In walking, various methodologies of inducing external perturbation have been studied and found to report adaptation, in the presence of the applied intervention, and de-adaptation, with the removal of applied intervention, in gait parameters (Bastian, 2008; Duschau-Wicke et al., 2009; Krishnan et al., 2012; Malone et al., 2012; Srivastava et al., 2016; Hidayah et al., 2018). The changes in the gait parameters reported in the current work were only present during the training session. Furthermore, during the training session, the changes were reflected immediately and were retained as long as the external resistive force was applied. The lack of adaptation during training and aftereffects during the post-training session in both the experiments implies a reactive response from the participants, and this did not yield motor learning in walking. This means that the participants compensated for the applied intervention through an abnormal gait pattern, demonstrating significant deviations.

The human lower limb musculoskeletal system, comprising of bones, muscles, tendons, joints, and tissues, reflects a complex, coupled dynamics. In particular, walking can be thought of as an integrated action of a mechanical serial-chain system of bones connected with joints and cyclical and coordinated actuation of muscles to apply joint torques to propel the body forward. Furthermore, there exist biarticular muscles in the leg that span over more than one joint, such as the hamstrings between the hip and knee and gastrocnemius between the knee and ankle. Notably, the actuation of a biarticular muscle induces multiple joint motions to further add to the coupled leg dynamics while walking (Zajac, 1993). Moreover, due to their uni-directional capability of force exertion, there exists redundancy in muscle actuators, which implies that a different set of muscles get activated synchronously to execute the leg motion during various phases of walking (Ivanenko et al., 2006; Chvatal and Ting, 2013). Thus, any notable change in an individual's walking pattern, which might have been necessitated because of incurred gait abnormalities or due to any applied mobility/force constraint, would require appropriate modulation and adjustments within the leg musculoskeletal system. Such modulations have been reported in various studies. For example, walking with higher ankle push-off in people with hip pain have been found to result in lower hip flexion movement (Lewis and Ferris, 2008). In contrast, due to the weakening of muscles with age, people have been reported to shift from distal to proximal muscles actuation to use predominantly uneconomical hip strategies (Mueller et al., 1994; Turns et al., 2007). Thus, the observation of a deviant gait pattern in the current work implies lower limb musculoskeletal adjustments to the applied resistance as if to compensate for a hip or an ankle joint abnormality.

The application of external resistance to the proximal hip joint or to the distal ankle joint in the two experiments reduced the perturbed leg's knee flexion values and ankle trajectory span during the swing phase. However, the two constraints necessitated distinct adjustments in the spatio-temporal gait parameters. In particular, temporal parameters, such as double support time, stride time, and stance time, reduced when the hip flexion was constrained in Experiment I; and spatio-temporal parameters, such as step length and stance time, reduced when the ankle plantarflexion was constrained in Experiment II. Cumulatively, these changes represent spatio-temporal gait asymmetry, which requires intralimb as well as interlimb coordination during walking. Locomotor adaptation studies that have been conducted in the literature provide ample evidence that an individual, depending on the applied perturbation, utilizes different adaptation mechanisms during lower limb coordination. For example, studies with a bilateral split-belt treadmill that induced spatio-temporal gait asymmetry have reported adaptation in the inter-limb parameters (Bastian, 2008; Malone et al., 2012). Studies with unilateral perturbations, such as with leg exoskeletons among others (Duschau-Wicke et al., 2009; Krishnan et al., 2012; Srivastava et al., 2016), have also reported adaptations in the intra-limb gait parameters. Notably, current results imply that gait abnormalities being proximal or distal may induce different lower limb coordination, and it would be effective to design a force intervention meant for deviant gait correction with such an understanding in mind.

Hemiparetic stroke patients have been observed to walk with a reduced paretic leg's foot trajectory in the sagittal plane of walking, essentially with a reduced area with shorter vertical and horizontal spans (Duschau-Wicke et al., 2009; Krishnan et al., 2012; Srivastava et al., 2016). A hemiparetic stroke patient may manifest multiple gait alterations, including multiple muscle weakness and spasticity (Carmo et al., 2012; Lauziere et al., 2014), but, interestingly, a single joint alteration in the current experiments resulted in seemingly similar deviations in the perturbed leg's foot trajectory. This is of interest, especially because some of the reported gait rehabilitation paradigms have successfully used robotic leg exoskeletons to apply external forces at multiple leg joints to achieve a desired foot trajectory performance by a hemiparetic stroke patient (Duschau-Wicke et al., 2009; Krishnan et al., 2012; Srivastava et al., 2016). It is most likely that, due to the coupled dynamics of the human lower limb musculoskeletal system, the effects of one joint abnormality, such as at the ankle or hip, on the foot trajectory are comparable to multiple joint abnormalities. Furthermore, it has been reported that healthy individuals do not demonstrate a unique coordination pattern of the hip, knee, and ankle joints to execute a desired change in the foot trajectory but can use multiple combinations to perform the desired change (Luu et al., 2017). Consequently, to a force intervention for gait correction, there exists the possibility that a human adapts the gait pattern to show improvement in overall walking measures but without any long-term improvement in the deviant parameters.

In a robotic rehabilitation paradigm, the use of a robotic exoskeleton is aimed at either assisting or resisting the deviant human lower limb joint motion to emulate a healthy gait (Banala et al., 2007; Duschau-Wicke et al., 2009; Krishnan et al., 2012; Hidayah et al., 2018). Typically, a task space approach is taken; the joint torque values required for a multi-link serial-chain model of a leg to execute a desired trajectory in the task space are computed. To apply the computed torque values on the human leg, external forces are applied by the robotic system at the leg segments, such as the thigh and shank. The human lower limb has redundant DOFs and is redundantly actuated through muscles, and there exist multiple ways of actuating muscles to achieve a particular task space performance. Thus, there can be cases where the musculoskeletal system adjusts undesirably to the applied robotic intervention. These aspects of human–robot interactions become very critical when considering a patient who has incurred multiple gait alterations and demonstrates a deviant gait. Markedly, Belda-Lois et al. (2011) notes that the effectiveness of a human–robot interaction in promoting motor learning depends on the imposed or self-selected actions. In this context, the current work has provided self-selected actions due to the use of controlled resistive forces applied at a single joint. These results with primitive force interventions can be used for validating musculoskeletal simulation studies and to develop effective subject-specific rehabilitation paradigms.

The majority of the leg exoskeletons used in the community are typically fully actuated, i.e., one actuator controls each degree of freedom, DOF, of leg motion. However, the consideration of mechanical design, cost, and size typically restrict the total DOFs of such robots. Some of the current systems allow passive mobility to compensate for undesirable constraints on human walking. In recent years, the use of wearable cable-driven systems has enabled the possibility of exerting a desired force without adding undesirable mobility constraints while minimizing the mass/inertia. The robotic platform used in the current work uses cables to apply the desired force intervention at a particular part of the leg segment. With the proposed controller to administer the gait phase adaptation during walking, the system can adapt to subject-specific gait frequency variations to apply the force at the desired point and during a desired phase of the gait cycle. Various studies in literature (Zhang et al., 2017; Bae et al., 2018; Ding et al., 2018) use the human-in-loop approach to adapt to subject-specific variations to provide suitable force intervention for gait assistance. In the current work, WeARS has been used to apply resistive force intervention at a single joint. In the future, studies with resistive and assistive force interventions will be conducted considering the single- and multi-joint movement of the lower limb to improve the walking performance of individuals with disabilities.

## 5. Conclusion

This work presents a cable-driven Wearable Adaptive Rehabilitation Suit (WeARS), which was used to implement single joint resistive force interventions at the hip and ankle joints separately. Two sets of experiments with eight healthy participants in each case were conducted. Experiment I involved the application of resistive forces on the posterior part of the thigh, and Experiment II involved the application of resistive forces on the anterior part of the foot. The resistive forces were applied from pre-swing to the terminal swing phase of walking to induce deviations in the gait pattern. The results of the two experiments reported that the healthy participants compensated for the applied intervention by adopting a deviant gait. Significant changes in the overall gait pattern were observed; in particular, reduction in joint range of motion and ankle trajectory in the sagittal plane of walking were reported, which required significant intralimb and interlimb adjustments. The observed results highlighted that a single-joint abnormality could result in abnormal gait characteristics, as observed in the case of multi-joint alterations. Furthermore, the results of the experiments reflected that a gait abnormality being distal or proximal can induce different spatio-temporal adaptation. In summary, the current work has explored self-selected actions due to controlled resistive forces applied at a single joint. Such an understanding of lower limb musculoskeletal adjustments to gait abnormalities are insightful to the adaptation of human–robot interactions during gait training. Thus, locomotor adaptations studies with primitive resistive force interventions are applicable when designing effective subject-specific rehabilitation paradigms.

## Data Availability Statement

The datasets generated for this study are available on request to the corresponding author.

## Ethics Statement

The studies involving human participants were reviewed and approved by IITGN Institutional Ethics Committee (IEC). The patients/participants provided their written informed consent to participate in this study. Written informed consent was obtained from the individual(s) for the publication of any potentially identifiable images or data included in this article.

## Author Contributions

SI contributed to the manuscript writing, fabrication of the system, controller design, data analysis, and experiments. JJ contributed to the manuscript writing and helped in conducting the experiments. VV conceived the presented idea and contributed to the manuscript writing, controller logic, system design, data analysis, and also supervised this work. All authors discussed the results and contributed to the final manuscript.

### Conflict of Interest

The authors declare that the research was conducted in the absence of any commercial or financial relationships that could be construed as a potential conflict of interest.
